# Influence of Liquid Nitrogen Pre-Freezing and Drying Methods on the Collagen Content, Physical Properties, and Flavor of Fish Swim Bladder

**DOI:** 10.3390/foods13172790

**Published:** 2024-09-01

**Authors:** Hongbing Dong, Jiwang Chen, Yujie Li, Chao Wang, Chuyi Jiao, Liuqing Wang

**Affiliations:** 1 Collage of Food Science and Technology, Wuhan Business University, Wuhan 430056, China; dhbin027@163.com; 2 Hubei Key Laboratory for Processing and Transformation of Agricultural Products, Wuhan Polytechnic University, Wuhan 430023, China; 3 College of Food Science and Engineering, Wuhan Polytechnic University, Wuhan 430023, China; 15027261451@163.com (Y.L.); wang1206ry@163.com (C.W.); 4 Enterprise-University Cooperative Innovation Center for Cryogenic Food Processing Technology Using Liquid Nitrogen, Wuhan Polytechnic University, Wuhan 430023, China; 18672667710@163.com

**Keywords:** fish swim bladder, Chinese longsnout catfish, drying, freeze-drying, liquid nitrogen, flavor

## Abstract

Fish swim bladder (FSB) is a type of traditional nutraceutical, but the lack of high-quality drying methods limits its premium market development. In order to obtain optimal-quality dried FSBs from Chinese longsnout catfish, the effects of liquid nitrogen pre-freezing (LNF) and drying on the physical properties and flavor of FSB were evaluated. Four methods were used for FSB drying, including natural air-drying (ND), hot-air-drying (HD), LNF combined with freeze-drying (LN-FD), and LNF combined with HD (LN-HD). Color, collagen content, rehydration ratio, textural properties, and flavor characteristics (by GC-IMS, E-nose, and E-tongue) were measured to clarify the differences among four dried FSBs. The results showed that ND cannot effectively remove moisture from FSB as the final product showed a stronger sourness in taste. HD led to a decrease in the collagen content and the collapse of the fiber structure in FSB. Compared to HD, LN-HD showed a higher collagen content (0.56 g/g) and a different flavor fingerprint. FSB treated by LN-FD had better physical qualities in terms of an attractive color, a high collagen content (0.79 g/g), low shrinkage, a higher rehydration ratio (2.85), and a soft texture, while also possessing richer characteristic flavors. The application of LN-FD may help the optimization of the nutrition level, rehydration ability, mouthfeel, and flavor of dried FSB.

## 1. Introduction

**Figure 1:**
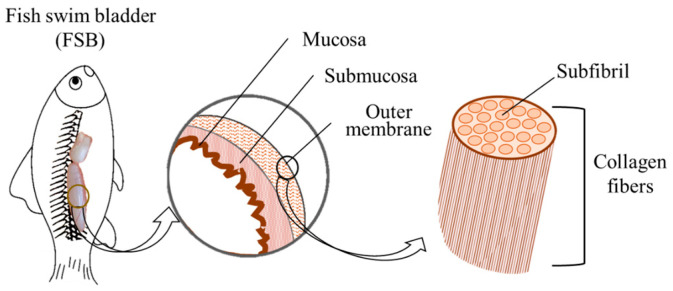
Schematic diagram of the collagen structure in the fish swim bladder.

The fish swim bladder (FSB) is a gas-filled organ found in most fish to control their buoyancy and swimming depth [1]. As shown in Figure 1, the FSB has a round sac shape with a semi-transparent milky white color, and it consists of three layers: the outer membrane, the submucosa, and the mucosa. The outer membrane is mainly composed of collagen fibers [2]. As a by-product of fish processing, the FSB is mainly consumed raw or as a dried product on the market [3]. Owing to its high collagen content and delicate taste, dried FSBs have been used as a type of tonic and a luxurious gourmet food in China and Southeast Asia for centuries [4]. Currently, the growing demand for healthy foods and nutraceuticals continues to promote the consumption of FSBs. Therefore, the high-quality processing and utilization of FSB has great significance for the further development of traditional fishery. In addition, most FSBs on the market come from marine fish, but the increasing demand and international trade involving FSBs will eventually present a threat to marine species [5]. Therefore, FSBs from freshwater fish have a certain economic and resource value. As a typical freshwater fish species, Chinese longsnout catfish (*Leiocassis longirostris*) can be considered a promising source of FSB.

Some studies have discussed the characteristics of collagen from the FSB and its utilization [6]. They confirmed that FSB collagen has potential for further applications in the food, cosmetic, and biomedical fields [7]. Li et al. [8] confirmed that the collagen extracted from FSB belonged to type I collagen and that the collagen sponge, with its fibrous structure, was a valuable bioactive material for various applications. However, only a few studies have explored the processing technologies of the FSB. Sethuraman et al. [9] investigated the effect of drying and frying conditions on the characteristics of seabass swim bladders, and their results showed that FSBs with a moisture content of 150 g/kg had the highest expansion ratio. It has been reported that the moisture content of FSBs from different freshwater fish is in the range of 67–74% [10,11]. The drying methods and the pretreatment before drying for FSB materials have a significant impact on the color, taste, and flavor properties, thereby affecting their market consumption and sales. Specifically, the structure of collagen is susceptible to changes due to its high sensitivity to environmental factors [12]. Thermal denaturation would, therefore, affect the mechanical properties and biostability of this collagen. Therefore, an appropriate drying method needs to be proposed to provide dried FSBs with high a collagen content and organoleptic quality, achieving these products’ high-value development in the market.

Natural air-drying (ND) and hot-air-drying (HD) are the common drying methods for FSB materials, but they have some disadvantages, such as longer treatment times, nutrient loss, and sensory quality deterioration [13]. Freeze-drying (FD) had been confirmed to be an effective approach to preserve heat-sensitive components in food, alleviate volumetric shrinkage, and optimize taste and aroma [14]. Cao et al. [15] found that the total essential amino acid content and α-helical structure of tilapia after FD were higher than with HD and microwave-drying, indicating the protection of proteins by FD. Before the FD process, a pre-freezing process is required to convert moisture into ice crystals. Generally, raw materials need to be frozen at −20 °C for about 24 h before FD, but the liquid nitrogen pre-freezing (LNF) method could shorten the frozen time to 5 min [16]. In addition, the small ice crystal formed during LNF may avoid the deformation of the tissue structure in frozen foods [17]. Addo et al. [18] studied the effects of pre-freezing (−80 °C) before FD and HD on the characteristics of hops and confirmed that low-temperature pre-freezing preserved the color and flavor molecules of hops. Hu et al. [19] demonstrated that LNF before the FD process could maintain the fiber length and original 3D reticular structure of a product while retaining more types of volatile compounds. In brief, LNF combined with the FD method might constitute a new method for obtaining high-quality dried FSBs. However, the practical effect of LNF and FD on the characteristics of FSBs is still unclear, affecting the development of high-quality FSB processing if not addressed.

This study aims to explore the influence of pre-freezing and drying methods on the collagen content, physical properties, and flavor of FSBs from Chinese longsnout catfish. The color, collagen content, textural properties, and flavor characteristics of FSB were compared and analyzed after using four drying methods, including ND, HD, LNF combined with FD, and LNF combined with HD. This work will give a more comprehensive understanding of the pre-freezing and drying methods for FSB.

## 2. Materials and Methods

### 2.1. FSB Preparation

In this study, FSBs of Chinese longsnout catfish were obtained from Yidanxian Agricultural Science & Technology Co., Ltd. (Yichang, Hubei Province, China). FSBs from the same batch with complete size and no pollution were chosen. Fresh FSBs were cut from the middle with scissors, and the outer oil film was torn off then cleaned. FSBs in a length range of 7–10 cm were selected and randomly divided into four groups for subsequent drying treatments.

### 2.2. Drying Methods for FSB

In this study, four drying approaches were utilized for FSB materials. All dried FSBs were attained when the weight became constant.

(1) ND: the fresh FSBs were placed on the surface of a tray in the sun under natural air conditions. FSBs were dried in Wuhan, Hubei Province, in October 2023. The temperature fluctuation was in the range of 24 ± 5 °C, while the relative humidity averaged 60 ± 5%. The process of ND for FSBs continued for approximately 3 days.
(2) HD: the fresh FSBs were set on the stainless-steel net plate in an oven equipped with a blower (HGZF-II-101-1, Yuejin Medical Equipment Co., Ltd., Shanghai, China). The temperature was set at 45 °C for 18 h, with an air velocity of 1.0 m/s [20].
(3) LNF combined with FD (LN-FD): the fresh FSBs were pre-frozen using liquid nitrogen machine (Cryogenic Science & Technology Co., Ltd., Beijing, China) under −80 °C for approximately 20 min to a central temperature of −18 °C. Pre-frozen FSBs were put in the vacuum freeze-dryer at a temperature of −40 °C and vacuum pressure of 40 Pa for 48 h.
(4) LNF combined with HD (LN-HD): the fresh FSBs were pre-frozen under −80 °C liquid nitrogen to a central temperature of −18 °C. Pre-frozen FSBs were put in an oven at 45 °C for 18 h. The hot-air-drying process of LN-HD is the same as that of HD FSBs, which can be used to evaluate the effect of pre-freezing treatment on drying.

These dried FSBs were packed in heat-sealed bags in a glass container to room temperature for further analysis.

### 2.3. Determination of Surface Color

The values of L* (lightness/darkness), a* (redness/greenness), and b* (yellowness/blueness) were determined using a colorimeter (CR-10, Shengguang, Co., Ltd., Suzhou, China) with illuminant D65, and the observation angle was 10°. The colorimeter was calibrated using a white and a black ceramic plate. The dried FSBs were cut into 15 mm sheets, then placed in a plastic dish for determination. The whiteness and yellowness of dried FSBs were calculated according to the method of Milovanovic et al. [21] based on the values of L*, a*, and b*. The results of each FSB were expressed as an average of ten measurements.

### 2.4. Determination of Proximate Composition

The moisture, lipid, and ash contents were determined as described by the Association of Official Analytical Chemists (AOAC) [22]. The moisture content was measured using the oven drying method (AOAC 952.08). Dried FSB (1.0 g) was placed in an aluminum box and heated to a constant weight in the oven at 105 °C. The moisture content was calculated by the mass difference before and after oven drying. The SZF-06C Soxhlet extraction system (Top Instrument Co., Ltd., Hangzhou, China) was used to analyze the lipid content (AOAC 948.15), which was calculated based on the weight of the extracted lipid. The ash content of dried FSBs was determined in a muffle furnace (SX-8-10, Tester Instrument Co., Ltd., Tianjin, China) at 550 °C (AOAC 938.08). The FSBs were repeatedly burned to constant weight, and the ash content was obtained by the weight of the measured ash. The most widely used method for the quantification of collagen is acid hydrolysis followed by a colorimetric hydroxyproline (Hyp) assay (AOAC 990.26). The collagen content of dried FSBs was determined by the colorimetric method [23]. Dried FSB samples were subjected to hydrolysis with 6 M HCl at 105 °C for 24 h, when collagen is transformed into Hyp. The Hyp in hydrolysate was oxidized to pyrrole in the presence of chloramine T, and the reaction with Ehrlich’s reagent generated substances that absorb in the visible region of the spectrum. The absorbance was recorded at 558 nm using UV spectrophotometry (UV-2100, Uniko Instrument Co., Ltd., Shanghai, China). The Hyp content obtained from the absorbance value was converted to collagen content by multiplying the corresponding collagen conversion coefficient.

### 2.5. Determination of Rehydration Ratio

The measurements of the rehydration curve and rehydration ratio for dried FSB were performed according to the method of Qiu et al. [24] with slight modifications. Briefly, dried FSBs were immersed in distilled water at 25 °C. The ratio of dried FSBs to water was 1:15 (*w*/*w*). During this process, the FSBs were taken at 2 min intervals for the first 10 min, and at 5 min intervals afterwards. The FSBs were then weighed for the calculation of rehydration ratio.

### 2.6. Texture Profile Analysis (TPA)

TPA was performed using the Texture Analyzer TA-XT (Stable Micro System, Surrey, UK) with P/50 probe. The tests were performed with 30% sample deformation at a pre-test speed of 2 mm/s, test speed of 1.0 mm/s, post-test speed of 2 mm/s, and trigger force of 5 g [25]. Hardness, springiness, brittleness, and chewiness of FSBs were determined from the force–displacement graph of the two compression/decompression cycles generated from TPA device.

### 2.7. Scanning Electron Microscope (SEM) Observation

The dried FSBs were cut into small cubes, then glued to the holder of a sputter coater. The dried FSBs were photographed using an S-4800 SEM (Hitachi, Tokyo, Japan) at an accelerating voltage of 80 kV.

### 2.8. Electronic Nose (E-Nose) Determination

The odor information of dried FSBs was collected and analyzed using the E-nose (CNose-28, Bosin Tech, Shanghai, China). The E-nose is equipped with 28 gas sensors to detect specific volatile compounds. Same weights of dried FSBs (0.5 g) were ground and placed in 20 mL sealed vials, then heated at 60 °C for 360 s for gas generation in headspace. The gas was pumped into E-nose equipment at 120 mL/min. The maximum output data were selected for analysis.

### 2.9. Gas Chromatography–Ion Mobility Spectrometry (GC-IMS) Determination

Information of volatile compounds in four dried FSBs was obtained using a Flavour Spec^®^ GC-IMS instrument (G.A.S., Dortmund, Germany) [26]. The dried FSBs were incubated for 10 min at 40 °C and then injected automatically in a splitless mode using an 85 °C syringe. The dried FSBs (2 g) were placed in a 20 mL headspace bottle. Headspace condition was set as 60 °C for 10 min, injection volume was 500 μL, and vibration speed was 500 r/min. The GC conditions included the following: FS-SE-54 quartz capillary column (15 m × 0.53 mm, 1 μm). The column temperature was 40 °C, carrier gas was N_2_, IMS temperature was 45 °C. Carrier gas flow rate program: Initial 2.0 mL/min for 2 min, linear increase to 10 mL/min for 8 min, to 100 mL/min for 10 min, and to 150 mL/min for 10 min.

### 2.10. Electronic Tongue (E-Tongue) Determination

Sourness, saltiness, richness, umami, aftertaste-A, aftertaste-B, astringency, and bitterness of FSBs were determined by E-tongue. The dried FSBs (1.0 g) were mixed with 180 mL of deionized water to extract the taste substances. The mixed solution was homogenized at 10,000 rpm for 30 s, then centrifuged at 4500 rpm and filtered for analysis. The supernatant (80 mL) was taken for E-tongue (TS-5000Z, INSENT, Fukuoka, Japan) determination. The collection time, stirring rate, and analysis time were set as 120 s, 60 rpm, and 30 s, respectively. The mean value of the three stable measurements was selected for the subsequent data analysis.

### 2.11. Statistical Data Analysis

All the data shown in this work were collected by at least three independent experiments. The results are expressed as mean ± SD. The significance of the differences was examined by one-way analysis of variance and Duncan′s multiple range tests with SPSS (Version 27.0; Chicago, IL, USA). *p* value < 0.05 was considered statistically significant. The data analysis was conducted using Origin 2023 (Origin Lab Corporation, Northampton, MA, USA).

## 3. Results and Discussion

### 3.1. Color Characteristics

Color directly affects the appearance of dried products and their market value. The effect of drying methods on the color of FSBs is shown in Table 1. It can be seen that FSBs treated by ND and HD had a transparent appearance, while LN-HD FSBs showed semi-transparent and yellow characteristics. Different from common FSBs in the market, LN-FD showed the appearance of milk, white and opaque. For the color determination results, LN-FD had a higher L* value (90.3), while LN-HD had a higher a* value (4.10) and b* value (13.54). According to the whiteness and yellowness index, LN-FD obtained the whitest FSBs, while HD and LN-HD had the yellowest. For the ND, HD, and LN-HD methods, FSBs were prone to oxidation due to prolonged exposure to an aerobic environment with high/ambient temperature, leading to a browning reaction caused by an oxidation reaction [27]. Maillard reactions during drying might also be the cause of the darkened appearance of FSBs owing to the presence of sugar, such as glucosamine [3,28]. Meanwhile, the cross-link between intermediates generated by lipid oxidation and proteins may cause protein denaturation, resulting in the color changes in FSBs [29]. The vacuum and low-temperature conditions of LN-FD effectively hindered the occurrence of browning, maintaining the original milky white of FSBs. However, there are few studies discussing the reasons for the color changes in dried FSBs. Some studies demonstrated that meat containing solidified fat with a high melting point appears whiter than when liquid fat with a lower melting point is present [30]. It is possible to speculate that the transparent appearance of dried FSBs may be due to the denaturation of low-melting-point fats induced by the ND and HD processes. Overall, LN-FD could effectively preserve the light color and original appearance of FSBs after moisture removal.

### 3.2. Proximate Composition Analysis

**Figure 2:**
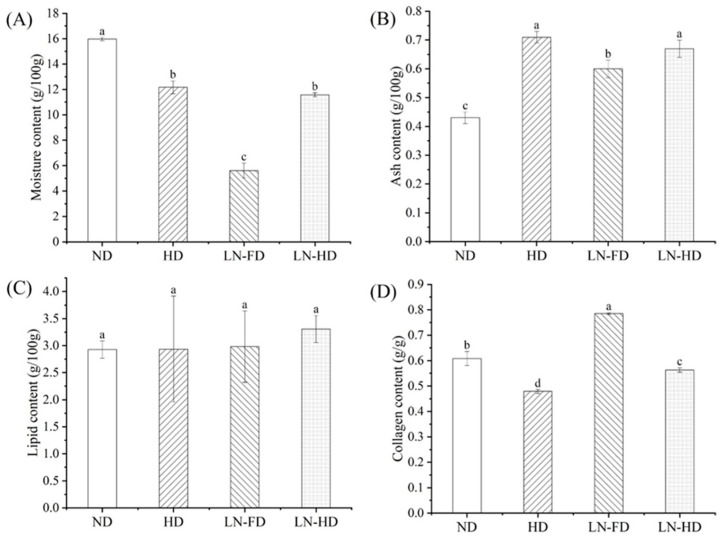
Changes in moisture content (**A**), ash content (**B**), lipid content (**C**) and collagen content (**D**) of fish swim bladder using different drying methods. ND represents natural air-drying, HD represents hot-air-drying, LN-FD represents liquid nitrogen pre-freezing combined with freeze-drying, and LN-HD represents liquid nitrogen pre-freezing combined with hot-air-drying. Within the same parameter, values with different letter are significantly different (*p* < 0.05).

The effect of pre-freezing and drying methods on the contents of moisture, ash, lipid, and collagen in FSBs is shown in Figure 2. The moisture content of four FSBs ranged from 5.61% to 15.97%. ND FSBs had the highest water content of 15.97%, indicating that water in FSBs could not be effectively removed by the ND process. Compared to other drying methods, LN-FD showed higher water removal and obtained the dried FSBs with 5.61% moisture. The HD and LN-HD methods can remove water to lower than 15%, but their moisture content was still higher than LN-FD FSBs. Compared to the process of ND and FD, HD with a higher drying rate could lead to the volumetric shrinkage and/or tissue collapse of FSBs. Liu et al. [31] observed a higher shrinkage curve of scallop adductors at a drying temperature of 45 °C than at 30 °C, which resulted in a slower drying rate curve. This alteration in FSBs led to the diffusive resistance in water movement through the boundary layer, making it difficult for internal moisture to diffuse and be removed.

Ash is an inorganic residue obtained after the complete ashing of FSBs. ND FSBs showed the lowest value of 0.43 g/100 g. ND had a longer time and slower drying process, leading to less impact on organic substances and relatively fewer residual ash substances. Moreover, HD FSBs have the highest ash content of 0.71 g/100 g, which could be due to that high-temperature conditions causing more chemical changes in organic matter. Ash from food products indicated the level of mineral content, which comprises crucial nutrients. Renuka et al. [32] reported that the mineral compositions of FSB from croaker mainly included K, Na, Ca, and Mg. The distribution of mineral elements and their solubility could also affect the ash content of FSBs [33]. 

Four dried FSBs showed a very low content of lipids (2.93–3.31%). There is no significant difference among the fat content of the four dried FSBs, indicating that the fat release was low in the pre-freezing and drying stages [34]. As the main ingredient in FSBs, the collagen content is an important indicator to evaluate the FSB quality. LN-FD FSBs had the highest collagen content of 0.79 g/g, which was significantly higher than other groups. This confirmed that LN-FD preserved collagen from the denaturation and loss during the drying process, which could have contributed to the drying temperature. Dong et al. [35] compared the physicochemical characteristics of collagen from four FSBs, and the results showed that the thermal denaturation temperature of collagen ranges from 29.8 °C to 38.6 °C. In addition, LF-HD had a higher collagen content than HD, indicating LNF partly prevented the loss of collagen during the HD process. Overall, LN-FD effectively removed moisture from FSBs and ensured a high content of collagen, suggesting the advantages of low-temperature drying.

### 3.3. Rehydration Curve and Rehydration Ratio Analysis

**Figure 3:**
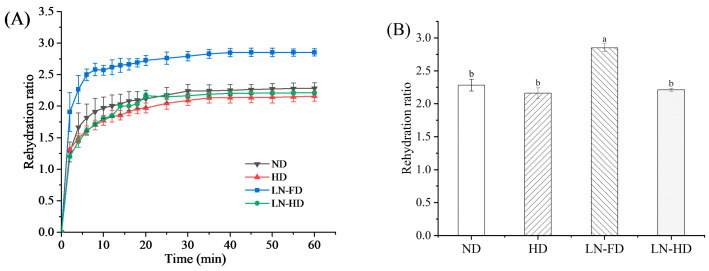
Rehydration curve (**A**) and rehydration ratio (**B**) of fish swim bladder using different drying methods. ND represents natural air-drying, HD represents hot-air-drying, LN-FD represents liquid nitrogen pre-freezing combined with freeze-drying, and LN-HD represents liquid nitrogen pre-freezing combined with hot-air-drying. Within the same parameter, values with different letter are significantly different (*p* < 0.05).

Generally, dried products are required to be rehydrated before their consumption; thus, ensuring a fast rehydration and high rehydration ratio is critical for the optimization of dried foods [36]. As shown in Figure 3, the observed trends in rehydration curves were a diffusion-controlled process. It can be seen that all rehydration curves showed a rapid increase in water absorption in the initial stage, followed by a gentle increase. In the first 10 min, LN-FD FSBs absorbed the most moisture, showing the highest rehydration rate. At the endpoint of the rehydration curve, LN-FD FSBs had the highest rehydration ratio of 2.85. For the FD process, the direct sublimation of small ice crystals would form a porous structure in FSBs under vacuum and low-temperature conditions. This structure could improve the rehydration ability of dried FSBs [37]. In addition, the other three rehydrated FSBs had no significant difference in the rehydration ability. This could be due to the collapsed and/or nonporous structure of FSBs after the ND, HD, and LF-HD processes. The reasons for the changes in the rehydration ability will be further explained based on structural changes during drying.

### 3.4. Textural Properties Analysis

TPA parameters are the crucial markers to evaluate the mouthfeel properties of food for consumers. In Table 2, it can be seen that there are significant differences in the hardness, brittleness, springiness, and chewiness of FSBs treated via four drying methods. These differences primarily contributed to the dehydration and shrinkage of materials, hard crust formation, and collagen denaturation and loss [38]. Among these textural properties, hardness represents the ability to resist external forces applied locally against its surface. The hardness of dried FSBs is ordered as LN-HD (6067 g) > HD (5112 g) > ND (4259 g) and LN-FD (3866 g), which has a consistent trend with that of rehydrated FSBs. The higher hardness of FSBs was possibly related to the occurrence of serious shrinkage and the formation of crust, which was mainly due to the quicker removal of moisture on the surface than that inside during the HD process [39]. LN-FD FSBs had a softer texture than others, which could be attributed to the formation of small crystals and their sublimation, resulting in a porous structure and loose surface. Moreover, the lower hardness of rehydrated LN-FD FSBs could also be attributed to the higher rehydration, which might provide a better mouthfeel for consumers. The chewiness is influenced by hardness, springiness, and cohesion, which affect the customer acceptance. Dried and rehydrated FSBs treated by LN-FD showed the lowest chewiness, while LN-HD had the highest chewiness. This was possibly owing to the loose and porous structure of FSBs caused by LN-FD, which is prone to irreversible deformation when subjected to external pressure. These results indicated that the textural properties of dried FSBs may be attributed more to the dehydration and contraction of the surface structure. Deng et al. [40] confirmed that FD could cause the most porous structure, softest texture, and highest rehydration of squid filets compared to the HD and heat-pump drying methods. In addition, the protein oxidation and denaturation may induce the formation of protein polymers, promoting the strengthening of the FSB structure [41]. Sinthusamran et al. [42] reported that the thermal transition temperature of FSB collagen from seabass was 35 °C, and the thermal denaturation generally occurs at a lower temperature. However, FSB is mainly composed of collagen; thus, its thermal denaturation trend could also be different from that of muscle tissue. The specific impact of FSB textural properties needs to be further analyzed in relation to their microstructure. Overall, FSBs treated by LN-FD were softer with lower chewiness, which is a promising product that differs from the traditional FSBs in terms of texture. 

### 3.5. Microstructure Analysis

**Figure 4:**
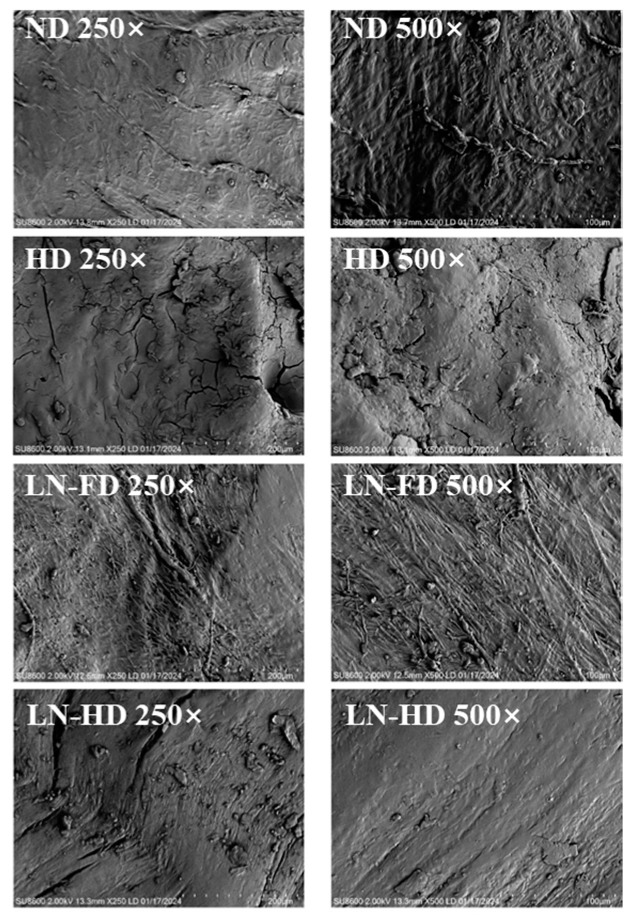
SEM images of fish swim bladder using different drying methods. ND represents natural air-drying, HD represents hot-air-drying, LN-FD represents liquid nitrogen pre-freezing combined with freeze-drying, and LN-HD represents liquid nitrogen pre-freezing combined with hot-air-drying.

The surface microstructure of four dried FSBs is shown in Figure 4. Samples were magnified to 500× and 250× for observation. FSB is mainly composed of regular collagen fibers, which are distributed with dense elastic fibers [43]. It can be seen that the surface of dried FSBs is a fibrous membrane layer. The surface of the ND FSBs showed tissue collapse due to dehydration shrinkage, while a relatively clear fibrous structure was observed. The structure of the HD FSBs is severely damaged, with cracks on the surface, and the collagen fiber structure could not be seen in the microstructure. This is because the of the rapid evaporation of surface water in the early stage of the HD process, which was consistent with the results of the rehydration ratio and texture. LN-FD FSBs had a clear and complete collagen fiber structure with more filament. With the simultaneous effect of the vacuum, the sublimation of small ice crystals during FD avoided the collapse of the surface structure [44]. Compared to HD, LN-HD showed a smoother surface with fewer cracks. This may be due to the combination of LNF and HD, which could be regarded as a freeze–thaw process, which could accelerate the whole drying process [45]. A higher drying rate may lead to the formation of harder crust in the following drying process, which could be the reason for the textural properties of LN-HD FSBs. Overall, compared to other drying methods, the good collagen structure of LN-FD FSBs could maintain the configuration of fiber, reduce textural degradation, and improve the rehydration ability, conserving the quality of FSBs.

### 3.6. Volatile Compounds Analysis

#### 3.6.1. E-Nose Analysis

**Figure 5:**
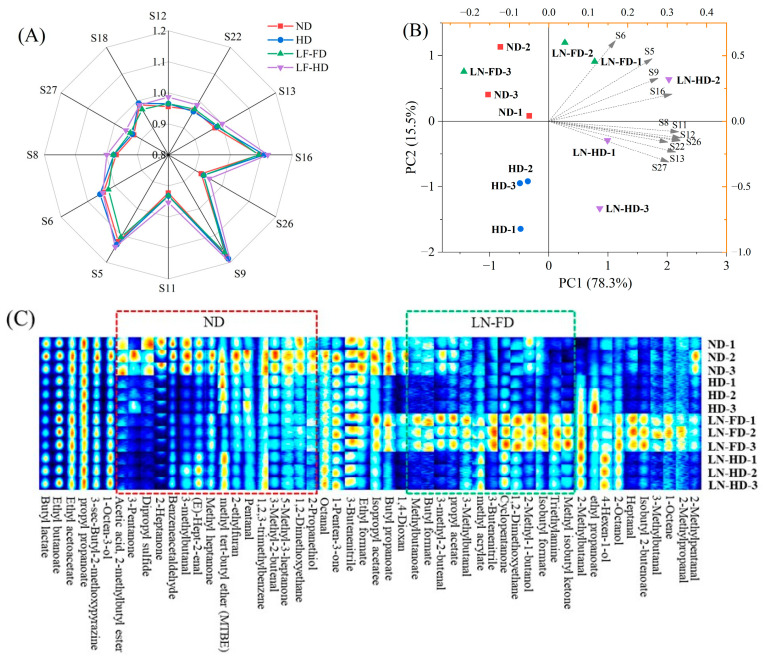
Radar diagram (**A**) and principal component analysis (**B**) from E-nose data, and dynamic fingerprints from GC-IMS data (**C**) of fish swim bladder using different drying methods. The color depth indicated the concentration of volatile compounds from GC-IMS data, red signal represents a higher concentration of compounds, blue signal represents a lower concentration of compounds. ND represents natural air-drying, HD represents hot-air-drying, LN-FD represents liquid nitrogen pre-freezing combined with freeze-drying, and LN-HD represents liquid nitrogen pre-freezing combined with hot-air-drying.

Various sensitive sensors were used to detect and differentiate the aroma in dried FSBs using four drying methods. [46]. In Figure 5A, certain sensors showed strong responses to the volatile compounds in four dried FSBs. Specifically, sensor S5 is sensitive to ammonia, S6/9 is sensitive to toluene and acetone, S8/12 represent the methane, S11 is sensitive to alkane, S16 is sensitive to H_2_S, S18 is sensitive to organic solvent (aromatic hydrocarbons, fatty hydrocarbons), S22 is sensitive to phenol and ketones, S26 is sensitive to esters and ketones, and S27 is sensitive to flue gas [47]. This suggested that dried FSBs contain a high abundance of toluene, alkanes, ketones, and other organic compounds. Compared to other drying methods, LN-HD had a higher concentration of methane, indicating the formation of volatile substances. This could be due to the thermal degradation and oxidation of protein/lipid in FSBs under high-temperature conditions, and the thaw during HD may have strengthened these reactions. Although all FSBs exhibited a low response to the S11/12/22/26 sensors, there were still variations in the response values among the different drying methods. In Figure 5B, the PCA results from E-nose are utilized to analyze the spatial distance and aroma distribution of four dried FSBs. PC1 and PC2 accounted for 78.3% and 15.5% of the total variance, respectively, and it can exhibit a significant portion of the odor information from FSBs. The distance between dried FSBs on a PCA plot can discriminate between how similar or different their odor is. Among four drying methods, ND and LN-FD FSBs had a similar odor but were significantly different from HD and LN-HD FSBs. The loading analysis can explain the magnitude of the contribution of each sensor to the FSB differentiation [48]. Sensors 5/6 contributed more significantly to varieties LN-FD than others, suggesting that LN-FD could induce higher levels of ammonia, toluene, and acetone in FSBs. These findings highlight the efficacy of four drying methods on the aroma denaturation of FSBs. However, the specific differences between these FSBs could not be comprehended solely using E-nose analysis, and further analysis needs to be combined with GC-IMS results.

#### 3.6.2. GC-IMS Analysis

To further understand the flavor changes in dried FSBs, volatile information from GC-IMS was obtained and analyzed. As displayed in Figure 5C, the spectrum of volatiles from four dried FSBs was obtained to establish a fingerprint for a comprehensive visualization comparison of flavor. Each row represents the signal peak of volatiles from the same FSBs, and each column represents the same volatile components from four drying methods. The color depth indicated the concentration of volatile compounds, and the red signal represents a higher concentration of compounds. Volatile compounds detected from GC-IMS are listed in Table 3. These compounds were classified into five types, including 5 alcohols, 13 aldehydes, 7 ketones, 14 esters, and other compounds. It is obvious that different drying methods can have a significant impact on the volatile compounds in FSBs. The signal peak of LN-FD was significantly higher than the other three drying methods, and the special flavor substances of LN-FD FSBs included 3-methylbutanal, 3-methyl-2-butenal, methyl isobutyl ketone, 1,2-dimethoxyethane, isobutyl formate and 2-methyl-1-butanol. Alcohol substances in FSBs could give aquatic products a mushroom-like odor, and aldehyde substances mainly result from the oxidation and degradation of lipids, forming a strong fat aroma [49]. LN-FD FSBs have stronger signals of alcohols and aldehydes that have a major contribution to aquatic products’ odor formation. Aldehydes could greatly contribute to the flavor of meat products owing to their high content and low threshold [50]. Overall, these substances could provide the special aroma of FSBs after LN-FD. Moreover, ND had more ketones that are mainly produced by the thermal oxidation or degradation of polyunsaturated fatty acids, contributing a distinctive aroma as a fruity flavor. Alkanes (C6–C19) have been identified in the volatiles of aquatic products, providing a specific aroma [51]. In addition, heterocyclic compounds such as pyrazine, pyridine, and pyrrole are characteristic products of the Maillard reaction. ND FSBs had a higher signal of pyrazine, which plays a role in the formation of the barbecue flavor and the generation of color [52]. Therefore, ND and LN-FD showed better aroma with more characteristic flavors of aquatic products, while the HD process removed some flavor organic substances.

### 3.7. E-Tongue Analysis

**Figure 6:**
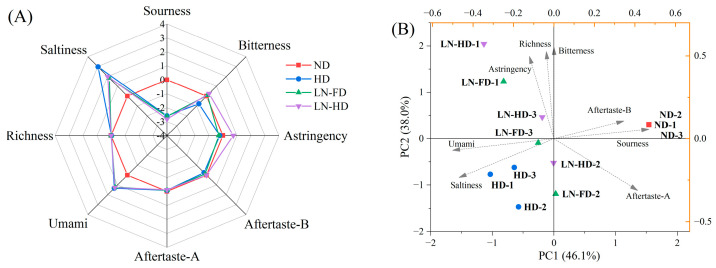
Radar diagram (**A**) and principal component analysis (**B**) from E-tongue data of fish swim bladder using different drying methods. ND represents natural air-drying, HD represents hot-air-drying, LN-FD represents liquid nitrogen pre-freezing combined with freeze-drying, and LN-HD represents liquid nitrogen pre-freezing combined with hot-air-drying.

To further differentiate the taste distinctions among four dried FSBs, the sourness, bitterness, astringency, umami, saltiness, aftertaste–astringency (aftertaste-A), aftertaste–bitterness (aftertaste-B), and richness were estimated using an E-tongue (Figure 6A). There were quite a few differences in richness, aftertaste-A, and aftertaste-A in FSBs by four drying methods. Compared to the others, ND showed lower saltiness and umami but higher sourness, indicating the degradation of umami amino acids and umami nucleotide in FSBs during the long-time ND process [53]. Umami molecules can modulate the sweet taste, enhance salty taste, and suppress bitterness, enhancing the overall gustatory experience [54]. HD and LN-FD can provide a high-quality taste profile for dried FSB with higher umami and lower astringency. Particularly, HD could weaken the bitterness and strengthen saltiness, which could be due to the fact that the high-temperature condition induced the degradation of bitter peptides in FSBs. The PCA analysis (Figure 6B) revealed that PC1 and PC2 accounted for a cumulative contribution rate of 84.1%, suggesting that the E-tongue data sufficiently represented enough taste information for four dried FSBs. PC1 shows a positive correlation with sourness and saltiness, while it has a negative correlation with umami and saltiness. PC2 exhibits a positive correlation with richness and astringency. The results indicated that ND had a significantly different taste profile than the other groups. HD FSBs exhibited relatively high levels of umami and saltiness but a lower level of sourness. The closeness of the distribution distance between LN-FD and LN-HD suggested a greater degree of taste similarity. Based on the comprehensive analysis of volatiles and non-volatiles in dried FSBs, ND retains its distinctive aroma but has a bitter and astringent taste. The HD process could provide more umami and saltiness in taste, but the high-temperature condition led to the loss of volatile odor. Overall, LN-FD was an optional method to retain better aroma and taste in dried FSBs.

## 4. Conclusions

Drying methods effectively affected the appearance, collagen, rehydration, texture, and flavor of FSBs. ND struggled to efficiently remove moisture from FSBs and showed stronger sourness in taste owing to long-time drying. HD induced a decrease in collagen content and the collapse of the fiber structure in FSBs, resulting in a lower rehydration ratio and a loss of flavor. LN-HD FSBs had similar physical properties with HD FSBs but had different flavor fingerprints. For LN-FD, the sublimation of small ice crystals in a vacuum environment maintained the skeleton structure and formed a loose porous structure in FSBs. Meanwhile, low-temperature drying conditions inhibited the oxidation and thermal denaturation of collagen. Overall, FSBs treated by LN-FD had a better physical quality of attractive color, low shrinkage, clear collagen fiber, and soft texture while also possessing richer characteristic flavors. This study provides a theoretical basis for choosing an appropriate drying method to produce high-quality dried FSBs. For the dried FSBs industry, non-thermal drying technologies such as FD can play a vital role in developing new products and expanding the traditional dried products market.

## Figures and Tables

**Table 1:** Color parameters of fish swim bladder using different drying methods. ND represents natural air-drying, HD represents hot-air-drying, LN-FD represents liquid nitrogen pre-freezing combined with freeze-drying, and LN-HD represents liquid nitrogen pre-freezing combined with hot-air-drying.

Samples	Appearance	L* Value	a* Value	b* Value	Whiteness Index	Yellowness Index
ND	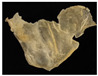	39.2 ± 3.56 ^bc^	1.12 ± 0.71 ^c^	9.65 ± 1.48 ^c^	38.4 ± 3.51 ^bc^	35.5 ± 6.32 ^b^
HD	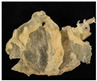	36.9 ± 3.60 ^c^	2.04 ± 0.48 ^b^	11.63 ± 0.81 ^b^	35.8 ± 3.59 ^c^	45.6 ± 7.31 ^a^
LN-FD	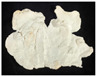	90.3 ± 3.03 ^a^	2.05 ± 0.64 ^b^	8.72 ± 1.17 ^c^	86.7 ± 2.63 ^a^	13.8 ± 1.96 ^c^
LN-HD	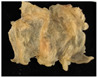	41.8 ± 2.90 ^b^	4.10 ± 0.92 ^a^	13.54 ± 1.91 ^a^	40.1 ± 2.76 ^b^	46.3 ± 6.32 ^a^

Note: Different letters in the same column indicate a statistically significant difference (*p* < 0.05).

**Table 2:** Changes in textual properties of dried fish swim bladder using different drying methods and their rehydrated (Re) samples. ND represents natural air-drying, HD represents hot-air-drying, LN-FD represents liquid nitrogen pre-freezing combined with freeze-drying, and LN-HD represents liquid nitrogen pre-freezing combined with hot-air-drying.

Samples	Hardness (g)	Brittleness	Springiness	Chewiness
ND	4259 ± 258 ^c^	4207 ± 301 ^c^	1.00 ± 0.00 ^a^	4787 ± 558 ^b^
HD	5112 ± 269 ^b^	5087 ± 303 ^b^	0.98 ± 0.03 ^a^	4878 ± 444 ^b^
LN-FD	3866 ± 592 ^c^	3865 ± 592 ^c^	0.91 ± 0.05 ^b^	3060 ± 614 ^c^
LN-HD	6067 ± 326 ^a^	6066 ± 325 ^a^	1.00 ± 0.00 ^a^	6130 ± 552 ^a^
Re-ND	3739 ± 251 ^ab^	3738 ± 251 ^a^	0.99 ± 0.01 ^a^	3524 ± 274 ^a^
Re-HD	3350 ± 372 ^ab^	3255 ± 534 ^a^	0.93 ± 0.08 ^a^	3371 ± 40 ^a^
Re-LN-FD	3189 ± 483 ^b^	3134 ± 455 ^a^	0.89 ± 0.04 ^a^	2659 ± 301 ^b^
Re-LN-HD	4066 ± 459 ^a^	4065 ± 459 ^a^	0.91 ± 0.04 ^a^	3547 ± 369 ^a^

Note: Different letters in the same column indicate a statistically significant difference (*p* < 0.05).

**Table 3:** Volatile compounds in dried fish swim bladder identified by GC-IMS.

Types	Compound	MW	RI	Rt (s)
Alcohols (5)	1-Octen-3-ol	128.2	1010.6	666.361
	2-Methyl-1-butanol	88.1	734.9	169.433
	2-Octanol	130.2	993.3	632.733
	4-Hexen-1-ol	100.2	881.6	394.778
	2-Propanethiol	76.2	593.3	56.881
Aldehydes (13)	(E)-Hept-2-enal	112.2	948.6	538.755
	Benzeneacetaldehyde	120.2	1052.7	742.829
	3-Methyl-2-butenal	84.1	787.1	229.59
	3-Methylbutanal	86.1	673.3	116.173
	3-methyl-2-butenal	84.1	733	167.551
	3-methylbutanal	86.1	640.6	91.272
	pentanal	86.1	682.7	123.69
	2-Methylbutanal	86.1	874	379.169
	Heptanal	114.2	883.5	398.568
	3-Methylbutanal	86.1	667.7	111.796
	2-Methylpropanal	72.1	598.4	60.548
	2-Methylpentanal	100.2	752.8	187.882
	octanal	128.2	1013.2	671.32
Ketones (7)	2-Heptanone	114.2	850.7	333.731
	Methyl heptenone	126.2	980	605.604
	cyclopentanone	84.1	788.6	231.673
	Methyl isobutyl ketone	100.2	734	168.522
	5-Methyl-3-heptanone	128.2	940.6	521.395
	1-Penten-3-one	84.1	685.6	126.039
	3-Pentanone	86.1	673.9	116.675
Esters (14)	Ethyl butanoate	116.2	789.6	232.941
	Acetic acid, 2-methylbutyl ester	130.2	876.1	383.472
	Ethyl acetoacetate	130.1	940	520.096
	Butyl formate	102.1	733.6	168.124
	Methylbutanoate	102.1	731.4	165.967
	Isobutyl formate	102.1	667	111.263
	propyl propanoate	116.2	804.1	254.113
	ethyl propanoate	102.1	700.7	138.456
	Isopropyl acetate	102.1	644	93.795
	Isobutyl 2-butenoate	142.2	993	631.958
	propyl acetate	102.1	990.6	627.245
	methyl acrylate	86.1	594.8	57.947
	Ethyl formate	74.1	622.7	78.133
	Butyl propanoate	130.2	880.3	392.112
Others (10)	3-sec-Butyl-2-methoxypyrazine	166.2	1091.6	809.63
	1,2,3-trimethylbenzene	120.2	979.8	605.13
	1,4-Dioxan	88.1	726.3	161.108
	2-ethylfuran	96.1	685.8	126.152
	1,2-Dimethoxyethane	90.1	636.3	88.07
	3-Butenenitrile	67.1	634.2	86.541
	1,2-Dimethoxyethane	90.1	672.4	115.485
	Triethylamine	101.2	669.3	113.061
	methyl tert-butyl ether (MTBE)	88.1	643.4	93.327
	Dipropyl sulfide	118.2	880.5	392.367

## Data Availability

The original contributions presented in the study are included in the article, further inquiries can be directed to the corresponding authors.
